# Age-dependent effects of H_2_S on post-traumatic stress disorder in adolescent and adult mice

**DOI:** 10.3389/fpsyt.2025.1546737

**Published:** 2025-06-09

**Authors:** Bing Gu, Ting Li, Haifen Zhao, Rui Yue, Qian Luo, Shuwen Yu, Tingting Li, Yijing Zhao, Dexiang Liu, Zhen Wang, Cyrus S. H. Ho

**Affiliations:** ^1^ Department of Medical Psychology and Ethics, School of Basic Medicine Sciences, Cheeloo College of Medicine, Shandong University, Jinan, Shandong, China; ^2^ Department of Psychiatry, Liaocheng People's Hospital, Liaocheng, Shandong, China; ^3^ Urology of Qilu Hospital, Shandong University, Jinan, Shandong, China; ^4^ Department of Physiology, School of Basic Medical Sciences, Cheeloo College of Medicine, Shandong University, Jinan, Shandong, China; ^5^ Department of Psychological Medicine, Yong Loo Lin School of Medicine, National University of Singapore, Singapore, Singapore

**Keywords:** post-traumatic stress disorder, H_2_S, CBS, inevitable foot shock, hippocampus

## Abstract

**Background:**

Among people with post-traumatic stress disorder (PTSD), there is an increased prevalence of age-related diseases. However, the biological mechanisms underlying this phenomenon remain incompletely understood.

**Methods:**

The expression of cystathionine β-synthase (CBS), one of the main enzymes for endogenous hydrogen sulfide (H_2_S) production in the brain, is age-dependent. In this study, we examined the influence of CBS/H_2_S on anxiety and depression-like behavior following the inescapable foot shock (IFS) procedure during early adolescence (postnatal days 28-35) or adulthood (postnatal days 63-70).

**Results:**

Our results showed that adult PTSD mice exhibited more pronounced decreases in H_2_S content and CBS expression in the hippocampus, which were associated with anxiety and depression-like behavior compared with adolescent PTSD mice. Administration of exogenous H_2_S significantly improved anxiety and depression-like behavior, mitigated synaptic plasticity deficits, and activated the CREB/BDNF signaling pathway in the hippocampus of adolescent PTSD mice. In addition, we found that high dose H_2_S could improve anxiety and depression-like behavior, mitigate synaptic plasticity deficits, and activate the CREB/BDNF signaling pathway, as well as increase H_2_S levels in the hippocampus. In contrast, injection of CBS antibody in the hippocampus of adult mice increased anxiety and depressive-like behavior.

**Conclusion:**

These results suggest that CBS/ H_2_S modulates PTSD-like behaviors in an age-dependent manner and may promote synaptic plasticity through activation of the CREB/BDNF pathway in the hippocampus of mice after IFS exposure.

## 1 Introduction

Post-traumatic stress disorder (PTSD) can develop following exposure to an event that involves actual or threatened death, serious injury, or sexual assault. Individuals suffering from PTSD typically experience symptoms of avoidance, intrusion and heightened arousal, as well as significant mood and cognitive changes (1). While many people experience traumatic events, the majority of trauma-exposed individuals do not develop PTSD. Some epidemiological studies suggest that age is one of the risk factors for fear-related disorders. For instance, the incidence of fear-related disorders tends to increase from adolescence to adulthood (2, 3). However, the biological mechanisms underlying this age-related susceptibility remain to be fully elucidated.

Hydrogen sulfide (H_2_S) is recognized as the third most common gasotransmitter following carbon monoxide and nitric oxide. Evidence indicates that endogenous H_2_S can be produced in various parts of the body, including the heart, blood and central nervous system (CNS). Endogenous H_2_S is synthesized from L-cysteine via three key enzymes: cystathionine β-synthase (CBS), cystathionine γ-lyase (CSE), and 3-mercaptopyruvate sulfur transferase (3-MST) (4, 5). CBS is particularly notable as the primary enzyme responsible for H_2_S production in the brain, with especially high expression especially in the hippocampus (6). H_2_S functions as a neuromodulator and plays a role in long-term hippocampal potentiation (7). Additionally, recent studies have demonstrated that H_2_S is involved in various physiological processes and pathological conditions, such as diabetes, ischemia, and depressive disorder (8). Moreover, H_2_S has been shown to exert antidepressant-like effects in mice exposed to chronic unpredictable mild stress (9, 10). NaHS is one of the exogenous donors of H_2_S, and NaHS can be hydrolyzed to Na^+^ and HS^-^, which can combine with H^+^ in the body to produce H_2_S, thereby increasing endogenous H_2_S levels (11). Therefore, NaHS is chosen as an exogenous donor of H_2_S in this study because of its stable H_2_S concentration in NaHS solution (11).

H_2_S levels can be significantly altered in age-related neurodegenerative diseases. For instance, brain H_2_S synthesis is severely decreased in Alzheimer’s disease patients, with reductions of up to 55%, and plasma H_2_S levels are negatively correlated with the severity of Alzheimer’s disease (12). Additionally, endogenous H_2_S levels decline with age in the hippocampus of adult and senescent mice, primarily due to impaired expression of H_2_S-generating enzymes such as CBS (13). In a severe 3×Tg-AD mouse model, continuous administration of sodium hydrosulfide, an H_2_S donor, for three months maintained or slowed brain pathology and subsequent cognitive deficits (14). These studies suggest that H_2_S and CBS play important roles in maintaining hippocampal function and cognitive ability, and their decreased expression levels may be associated with age-related cognitive dysfunction. Despite these findings, the relationship between endogenous H_2_S production and age-differences in PTSD process remains unclear. Furthermore, the potential protective effects of H_2_S supplementation against age-related behavioral alterations are not well understood. To address these gaps, we investigated the effect of inescapable foot shock (IFS) exposure during adolescent and adulthood on H_2_S levels in the hippocampus. We also explored the underlying mechanisms of H_2_S action by examining hippocampal synaptic plasticity.

## 2 Methods and materials

### 2.1 Study ethics declarations

All studies were conducted with governmental approval and adhered to the NIH guidelines for the care and use of laboratory animals. The experimental procedures followed both the ARRIVE and STAIR guidelines. Additionally, the study protocols were approved by the Animal Ethics Committee of Shandong University (ECSBMSSDU2022-2-83) and were in full compliance with their guidelines.

### 2.2 Animals and drug treatment

Adolescent (postnatal day 21) and adult male C57BL/6 mice (postnatal day 56) were obtained from the Experimental Animal Center of Shandong University. Mice were housed in a 12-hour dark/light cycle with unrestricted water and food. The ambient temperature was controlled at 20 ± 2°C and humidity at 60-70%. In this study, mice were supplied with endogenous H_2_S by intraperitoneal injection of NaHS. Based on a large body of previous literature, the dose of intraperitoneal NaHS was 5.6 mg/kg/d, a dose that has been validated by numerous studies for its efficacy and safety in animal models of stress-related diseases (15–17). The selection of 5.6 mg/kg/d NaHS dose was further validated through body surface area (BSA) normalization from established rodent-to-human conversion protocol (18, 19). Based on the standard BSA ratio (mouse:human = 12.3:1), our experimental dose translates to: Human equivalent dose = 5.6 mg/kg/12.3​≈0.45 mg/kg. It was shown that, similar to rats, the average free H_2_S content in mammalian brains was about 50-160 μM (20). Based on a standard human body weight of 70 kg, this administered dose of H_2_S achieves an approximate systemic concentration of 16.5 μM. This falls within the clinically relevant range for H_2_S donors in preliminary human trials.

### 2.3 IFS procedure

The PTSD model was established using a modified version of a previously described procedure (21). Mice were placed in a closed electric shock chamber, where their feet were subjected to consecutive, unavoidable shocks delivered in a semi-random manner, for a total of 18 shocks. The intervals between shocks were randomly determined and ranged from 30 and 120 seconds. IFS procedure was conducted for 7 days, with two sessions per day, and the interval between the two daily sessions was no less than 4 hours. To prevent interference from environmental factors, mice in the control group were placed in a similar chamber that was shielded from light but did not deliver electric shocks.

### 2.4 Experimental design

I. To investigate the difference in PTSD-like behavior following IFS exposure between early adolescence and young adulthood, mice were randomly assigned into four groups after a 7-day acclimatization period: adolescent control group (Ado+Control), adult control group (Adu+Control), adolescent IFS group (Ado+IFS), and adult IFS group (Adu+IFS). Mice in the Ado+IFS and Adu+IFS groups underwent the IFS procedure for 7 days, while those in the Ado+Control and Adu+Control groups remained untreated during this period.

II. To investigate the effects of NaHS treatment on PTSD-like behavior following IFS exposure, mice were randomly assigned into four groups: adolescent IFS group (Ado+IFS+PBS), adolescent IFS group receiving NaHS treatment (Ado+IFS+NaHS), adult IFS group (Adu+IFS+PBS), and adult IFS group receiving NaHS treatment (Adu+IFS+NaHS). Mice in the Ado+IFS+NaHS and Adu+IFS+NaHS groups received NaHS (5.6 mg/kg/d) via intraperitoneal injection (i.p.) 30 minutes after the shocks, while the mice in the Ado+IFS and Adu+IFS groups received an equivalent volume of PBS 30 minutes after the shocks. NaHS was dissolved in PBS, and the dose of NaHS was selected based on a previous study (22).

III. To achieve optimal outcomes, adult PTSD mice were administered two doses of exogenous H_2_S. Mice were randomly assigned to two groups: the adult IFS group treated with PBS (Adu+PBS+IFS+PBS), and the adult IFS group receiving two doses of NaHS treatment (Adu+NaHS+IFS+NaHS). Mice in the Adu+NaHS+IFS+NaHS group received NaHS (i.p.) 30 minutes before the shocks and again 30 minutes after the shock. In contrast, mice in the Adu+PBS+IFS+PBS group received PBS (i.p.) at the same time points.

IV. To investigate the role of the H_2_S/CBS in PTSD-like behavior, mice were randomly assigned into two groups: adult mice receiving IgG (Adult+IgG) and adult mice receiving the CBS antibody (neutralizing CBS) (Adult+CBS antibody).

### 2.5 Behavioral tests

#### 2.5.1 Conditioned fear test

To assess contextual freezing, mice were placed back into the same shock box used previously, and their spontaneous activity was observed and recorded for 5 minutes. Subsequently, 1-minute auditory cue was presented to investigate cue-freezing. The mice were then placed in a contextually similar chamber for 5 minutes, and the duration of freezing behavior (defined as the absence of movement except for breathing) was recorded.

#### 2.5.2 Open field test

Mice were placed in the center of a wooden open-field box (40 cm × 40 cm × 40 cm) and allowed to explore freely for 5 minutes. The total distance traveled and time spent in the central square were recorded using a SMART video tracking system (SMART 2.5, Panlab, Cambridge, USA). To eliminate any olfactory cues left by the previous mouse, the floor and walls of the open-field apparatus were thoroughly cleaned with 75% ethanol after each test.

#### 2.5.3 Tail suspension test

The tail suspension test (TST) was used to assess depressive-like behavior in mice. Each mouse was suspended by the tip of its tail using a magnet in a rectangular box. The test duration was 6 minutes, with the first 2 minutes allowing the mice to acclimatize, and the last 4 minutes used to record immobility (resting) times.

#### 2.5.4 Forced swimming test

The forced swimming test (FST) is used to assess despair-like behavior in animals. Each mouse was placed in a transparent cylindrical container approximately 45 cm in height, filled with water maintained at 23 ± 1°C. The test duration was 6 minutes, with the first 2 minutes allocated for acclimatization and the remaining 4 minutes used to record the immobility time of the mice in the water.

### 2.6 Western blot analysis

Total protein was extracted using radio immunoprecipitation assay lysis buffer containing phenylmethylsulfonyl fluoride solution (Solarbio, Beijing, China) and phosphatase inhibitor (Servicebio, Wuhan, China) at a mixing ratio of 100:1:1. The lysates were then centrifuged at 12,000g for 10 minutes at 4°C to obtain the protein supernatant. Protein concentration was determined using a BCA assay kit. Subsequently, the proteins were separated by SDS-PAGE electrophoresis and transferred onto PVDF membranes. The membranes were blocked with 10% skim milk for 1 hour at room temperature and then incubated with primary antibodies (**Supplementary Table S1**) overnight at 4°C. After three washes with TBST, the membrane was incubated with the secondary antibodies for 1 hour at room temperature. The protein bands were visualized using a chemiluminescence detection system, and the intensities of the bands were quantified using ImageJ software (NIH, Scion Corporation, Frederick, MD). Protein bands data were processed using the normalization method. The raw band gray values of each group of target proteins were first normalized to the corresponding β-actin in the same lane. Normalized value = (target protein gray value)/(β-actin gray value). Next, the normalized values of all comparison group samples were averaged to establish a baseline. The normalized values of all experimental groups were then divided by this comparison group mean to determine relative protein expression. Final protein levels were expressed as fold changes relative to the comparison group (set as 1.0). Fold change = (normalized target value for treatment group)/(average normalized value for comparison group).

### 2.7 Golgi staining

After the mice were sacrificed, the brains from each group were placed in 4% paraformaldehyde fixative for at least 24 hours and then stained with a Golgi staining kit. Sholl analysis was performed using ImageJ 6.0 software. The hippocampal cornu ammonis 1 (CA1) and dentate gyrus (DG) regions were analyzed, with six neurons selected from each region. The intersections of neuronal dendrites with concentric circles were counted within a 200× field of view. Additionally, the density of dendritic spines was quantified within a 1000× field of view.

### 2.8 Stereotactic injection of CBS antibody and IgG

Mice were anesthetized with isoflurane and secured in a stereotaxic apparatus. Small burr holes were drilled on the skull, posterior 2.06 mm posterior to the bregma and 1.5 mm lateral to the midline. A volume of 1 μL of either CBS antibody or IgG was injected at a rate of 0.1 μL/min at a depth of 1.8 mm (23). Following the injection, the microinjector was left in place for 10 minutes before being slowly retracted. Behavioral tests were conducted 3 days after the injection of CBS antibody or IgG. Subsequently, the mice were sacrificed for further experiments.

### 2.9 Measurement of H_2_S production

The traditional methylene blue method was used to quantify H_2_S levels. Briefly, hippocampus tissue was homogenized and incubated with zinc acetate, followed by reaction with *N,N-dimethyl-p*-phenylenediamine sulfate. The absorbance was measured at 670 nm, and the H_2_S concentration was calculated using a NaHS calibration curve.

### 2.10 Hematoxylin and eosin staining

Mice were perfused to remove the heart, liver, spleen, lung, and kidney. These five organs were then fixed in 4% paraformaldehyde for 48 hours, followed by processing into paraffin sections. After deparaffinization, the nuclei were stained with hematoxylin, and the cytoplasm was stained with eosin. The morphology of the organs was subsequently examined under light microscopy.

### 2.11 Statistical analysis

All data are expressed as the mean ± SD. Behavioral data were analyzed using a two-way analysis of variance (ANOVA), with the IFS exposure and age as fixed factors. One-way ANOVA was used for multiple comparisons, followed by the Bonferroni *post hoc* test for significant effects. Differences between two groups were assessed using the independent samples t-test (two-tailed). For data that did not fit a normal distribution, the rank sum test was used. Correlation was measured using Pearson correlation analysis. The sample size was determined based on a pilot study to identify the minimum number of mice required to detect significant differences in the behavioral experiment between the control and IFS groups. Using a power analysis (http://powerandsamplesize.com/Calculators/), we estimated that a sample size of 7 mice per group was required to detect a difference with a type I error (α) of 0.05 and a power of 0.8. Based on this calculation, at least 7 mice were allocated to each experimental group to assess the behavioral performance. All statistical analyses were performed using SPSS 25.0 software. Differences were considered significant when the *p* value was < 0.05.

## 3 Results

### 3.1 H_2_S and CBS expression were decreased in the hippocampus of adult PTSD mice, associated with anxiety and depression-like behavior

**Figure 1:**
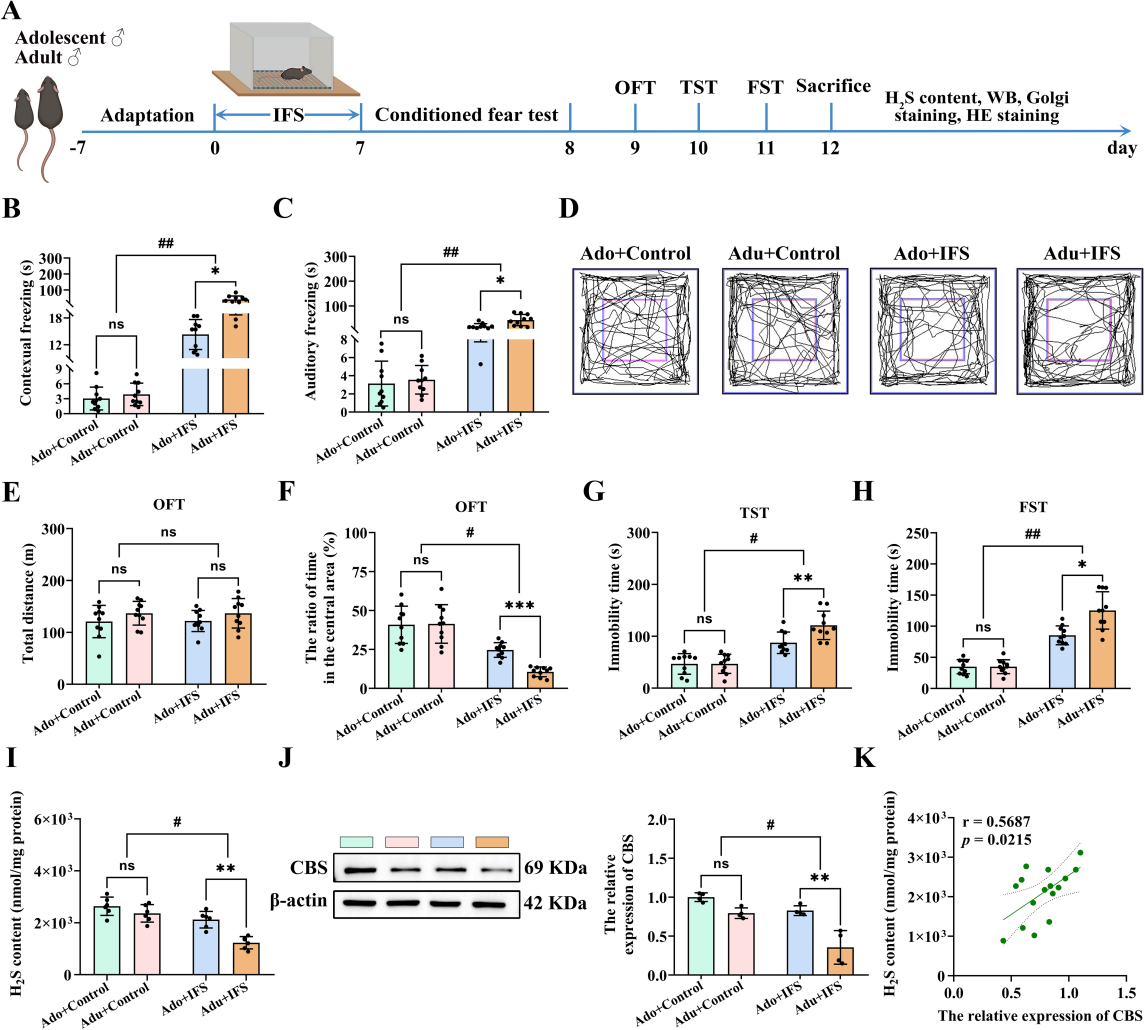
Adult PTSD mice had more pronounced decrease in H_2_S content and CBS expression in the hippocampus, associated with greater anxiety and depression-like behavior. **(A)** Experimental paradigm for mice exposed to inescapable foot shock (IFS) during adolescence or adulthood. After the last day of IFS, the conditioned fear test was performed on the first day, open field test (OFT) was performed on the second day, tail suspension test (TST) was performed on the third day and forced swimming test (FST) was performed on the fourth day. After mice were sacrificed, the H_2_S content in the hippocampus was tested, and follow-up tests were performed. **(B)** The freezing time in the contextual freezing test. **(C)** The freezing time in the cue freezing test. **(D)** The sample travel pathway in the OFT. **(E)** The total distance in the OFT. **(F)** The ratio of time in the central area in the OFT. **(G)** The immobility time in the TST. **(H)** The immobility time in the FST. **(I)** Detection of H_2_S content in the hippocampus of mice. **(J)** Western blot analysis of CBS expression in the hippocampus. **(K)** Correlation analysis of H_2_S content and CBS expression in the hippocampus. N=10 per group in A-H, N=6 per group in I, N=4 per group in **(J)** The data were presented as mean ± SD, ns indicates no significant differences, *p<0.05, **p<0.01, ***p<0.001 according to independent samples *t*-test (Ado+Control and Ado+IFS group as a comparison group), ^#^ *p*<0.05, ^##^ *p*<0.01 according to two-way analysis of variance (ANOVA) followed by Bonferroni’s *post hoc* testing in **(B-J)**, and correlation analysis in **(K)**.

The experimental paradigm of IFS was illustrated in **Figure 1A**. To verify the effect of a plantar electric shock on conditioned fear memory, mice were first assessed on the second day of IFS using the conditioned fear test to measure freezing time. Two-way ANOVA revealed significant interactions between IFS exposure and age for contextual freezing [F(1,36)=12.956, *p*<0.01] and cue freezing [F(1,36)=9.744, *p*<0.01]. Mice exposed to IFS exhibited significantly longer freezing times in both the contextual fear test (*p*<0.01) and cue fear tests (*p*<0.01) compared to the controls. Additionally, the Adu+IFS group demonstrated longer freezing times in both the contextual fear test (*p*<0.05) and cue fear test (*p*<0.05) compared to the Ado+IFS group (**Figures 1B, C**).

To verify the IFS-induced differences in anxiety-like behavior between adolescence and adult mice, OFT was conducted. Anxiety-like behavior was assessed by measuring the time spent in the central region of the open field (**Figure 1D**). A trajectory diagram illustrating the movement paths of the mice was also shown in **Figure 1D**. To ensure the results were not confounded by overall mobility due to IFS exposure, the total distance traveled by the mice was measured. No significant differences were observed in the total distance traveled among the groups (**Figure 1E**). IFS and age had significant effects on the time taken by the mice to enter the central region. Compared with the control group, mice exposed to IFS spent significantly less time dwelling in the central zone (*p*<0.05), and mice in the Adu+IFS group spent a shorter time dwelling in the central zone compared with the Ado+IFS group (*p*<0.001, **Figure 1F**).

To assess IFS-induced differences in depression-like behavior, TST and FST tests were conducted in both adolescence and adult mice. Two-way ANOVA revealed significant interaction between IFS exposure and age in both the TST [F(1,36)=5.892, *p*<0.05; **Figure 1G**] and FST [F(1,36)=11.612, *p*<0.01; **Figure 1H**]. Furthermore, after exposure to IFS, adult mice showed significantly longer immobility time than adolescence mice (*p*<0.01). Similarly, immobility time in the FST was significantly increased in mice exposed to IFS, with adult mice displaying significantly longer immobility times than adolescence mice (*p*<0.05).

H_2_S contents in the hippocampus of mice exposed to IFS was significantly decreased compared to the control group (*p*<0.05). The decrease of H_2_S content was more pronounced in adult mice compared to adolescent mice (*p*<0.01; **Figure 1I**). Western blot analysis revealed that the CBS expression in the hippocampus of IFS-exposed mice was significantly reduced compared to controls (*p*<0.05). This reduction was more significant in adult mice compared to adolescence mice (*p*<0.01; **Figure 1J**). Correlation analysis showed a positive correlation between H_2_S content and CBS expression in mice hippocampus (**Figure 1K**). H&E staining indicated that IFS exposure did not induce toxicity in the brain or major peripheral organs (heart, liver, spleen, lungs and kidneys) (**Supplementary Figure S1**).

### 3.2 PTSD-induced deficits in hippocampal synaptic plasticity were associated with suppressed CREB phosphorylation and BDNF expression in adult mice

**Figure 2:**
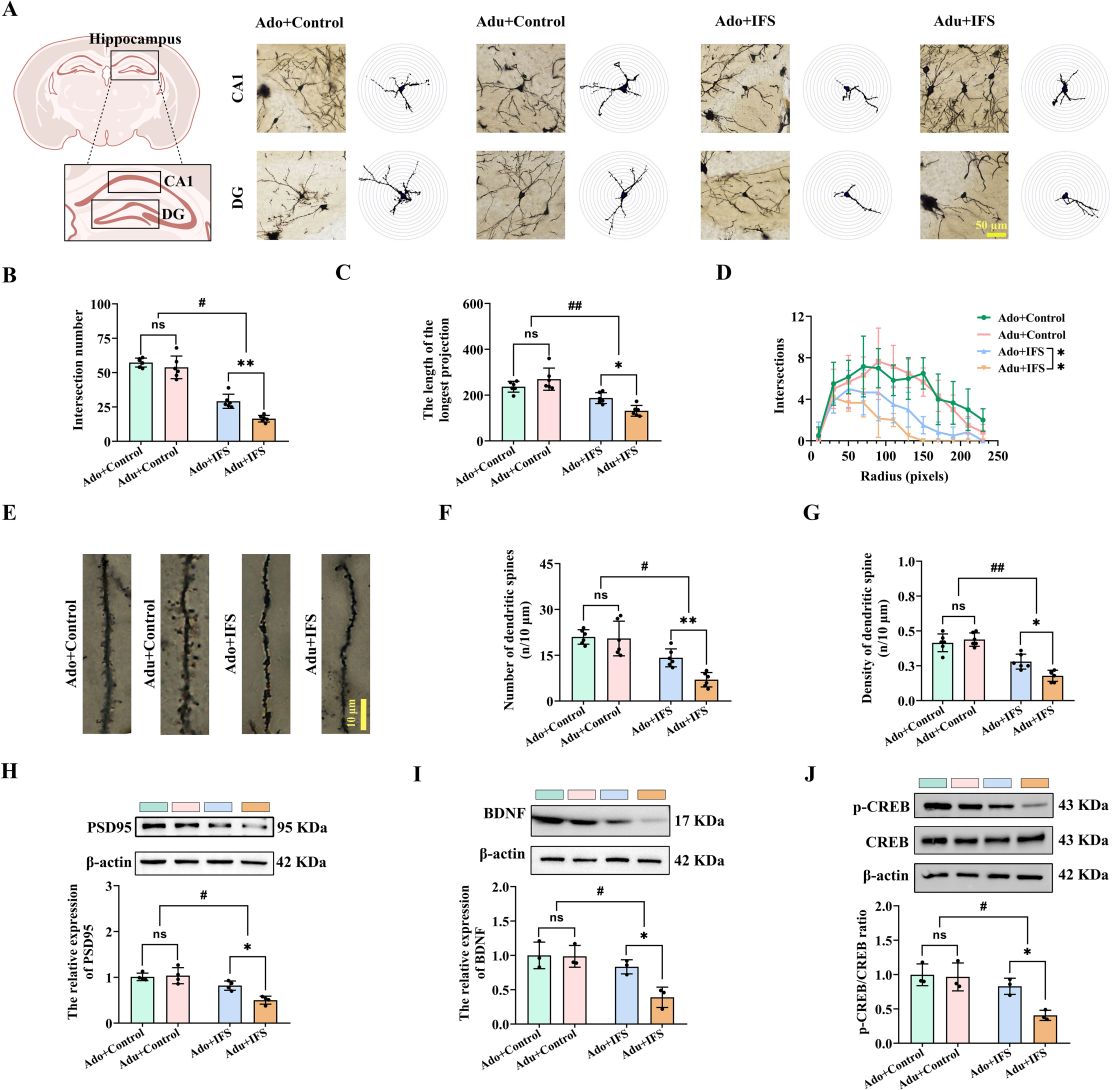
Adult PTSD mice had pronounced decrease in hippocampal synaptic plasticity, associated with less p-CREB/CREB and BDNF expression. **(A)** Brain atlas showed the locations of CA1 and DG, as well as Golgi staining in these two regions of hippocampus. Scale bar=50 μm. **(B)** The number of intersections of dendritic branches in the hippocampus of adolescent and adult mice. **(C)** The longest length of dendritic branches in the hippocampus of adolescent and adult mice. **(D)** Line graphs of dendritic branch lengths in adolescent and adult mice. **(E)** Golgi staining of dendritic spines in the hippocampus of adolescent and adult mice. Scale bars=10 μm. **(F)** Number of dendritic spines in the hippocampus of adolescent and adult mice. **(G)** Density of dendritic spines in the hippocampus of adolescent and adult mice. **(H)** Western blot analysis of PSD95 in the hippocampus. **(I)** Western blot analysis of BDNF in the hippocampus. **(J)** Western blot analysis of p-CREB and CREB in the hippocampus. N=6 per group in **(A–C, F, G)**; N=4 per group in **(H)**; N=3 per group in I and **(J)** The data were presented as mean ± SD, ns indicates no significant differences, *p<0.05, **p<0.01, according to independent samples *t*-test (Ado+Control and Ado+IFS group as a comparison group), ^#^ *p*<0.05, ^##^ *p*<0.01 according to two-way ANOVA followed by Bonferroni’s *post hoc* testing in **(B–D, F–J)**.

Changes in the hippocampal neuronal structure are crucial in the onset and development of cognitive and psychiatric disorders (24). Therefore, we analyzed the dendritic complexity and spine density of CA1 and DG neurons, as well as pyramidal cells in the four groups using Golgi staining (**Figure 2A**). The total number of intersections indicates the richness of neuronal branching, with more branches and longer lengths indicating better neuronal growth (22). Two-way ANOVA revealed significant interactions between IFS and age for both the number of dendritic branch intersections [F(1,20)=4.563, *p*<0.05] and the length of dendritic branches [F(1,20)=11.647, *p*<0.01]. Reductions in the number of intersections (*p*<0.01) and the longest length (*p*<0.05) of dendritic branches were more pronounced in the Adu+IFS group compared to the Ado+IFS group (**Figures 2B, C**). The number of intersections within 250 pixels is shown in a line graph (**Figure 2D**). Additionally, we analyzed dendritic spines in the hippocampus of adolescence and adult mice using Golgi staining (**Figure 2E**). Results showed that dendritic spine number (*p*<0.05) and density (*p*<0.01) were significantly reduced in IFS-exposed mice compared to controls. These reductions were more pronounced in the Adu+IFS group compared to the Ado+IFS group (*p*<0.01 for spine number; *p*<0.05 for spine density; **Figures 2F, G**). PSD95, a key postsynaptic density (PSD) protein, involved in synaptic development and plasticity (25), was significantly downregulated in IFS-exposed mice (*p*<0.05). Moreover, the Adu+IFS group exhibited a more pronounced decrease in PSD95 expression compared to the Ado+IFS group (*p*<0.05; **Figure 2H**).

The CREB/BDNF signaling pathway has been implicated in regulating several functions, including cell survival, synaptic structure, and synaptic plasticity (26). Two-way ANOVA revealed a significant interaction of the expression of BDNF [F(1,8)=5.841, *p*<0.05; **Figure 2I**] and p-CREB/CREB [F(1,8)=5.409, *p*<0.05; **Figure 2J**] in the hippocampus of both adolescence and adult mice. IFS exposure reduced the expression of p-CREB/CREB and BDNF in the hippocampus of both the Ado+IFS and Adu+IFS groups. Notably, the reduction in p-CREB/CREB (*p*<0.05) and BDNF (*p*<0.05) expression were more pronounced in the Adu+IFS group compared to the Ado+IFS group.

### 3.3 NaHS treatment increased CBS/H_2_S levels in the hippocampus of adolescent PTSD mice and improved anxiety and depression-like behavior

**Figure 3:**
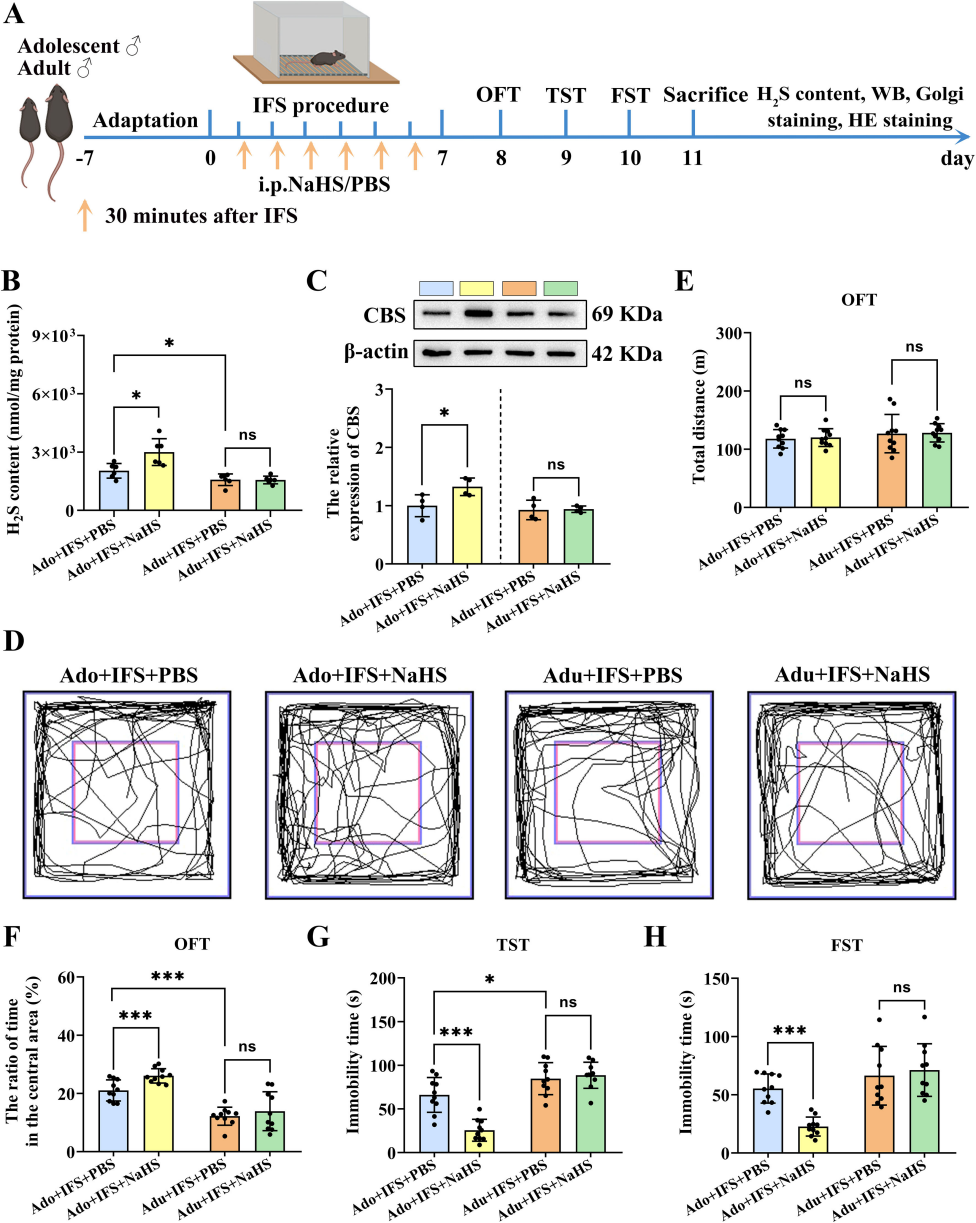
NaHS treatment increased CBS/H_2_S levels in the hippocampus of adolescent PTSD mice and improved anxiety and depression-like behavior. **(A)** Experimental flow chart. The mice in the Ado+IFS+NaHS and Adu+IFS+NaHS groups were administered NaHS (5.6 mg/kg/d) by intraperitoneal injections (i.p.) 30 min after the shocks, while mice in the Ado+IFS and Adu+IFS groups received PBS 30 minutes after the shocks. After completing IFS, OFT was performed on the first day, TST was performed on the second day and FST was performed on the third day. After mice were sacrificed, the H_2_S content in the hippocampus was tested, and follow-up tests were performed. **(B)** Detection of H_2_S content in the hippocampus of mouse. **(C)** Western blot analysis of CBS in the hippocampus. **(D)** The sample travel pathway in the OFT. **(E)** The total distance in the OFT. **(F)** The ratio of time in the central area in OFT. **(G)**The immobility time in the TST. **(H)** The immobility time in the FST on the third day post-IFS. N=6 per group in **(B)**, N=4 per group in **(C)**, N=10 per group in **(D–H)**. The data were presented as mean ± SD, ns indicates no significant differences, *p<0.05, ***p<0.001 according to independent samples *t*-test (Ado+IFS+PBS and Adu+IFS+PBS group as a comparison group) in **(B–E, F–H)**.

To investigate whether the decreased H_2_S levels in the hippocampus after exposure to IFS led to anxiety and depression-like behavior, mice were administered NaHS to observe subsequent behavior change (**Figure 3A**). Results showed that H_2_S in the hippocampus was significantly increased in the Ado+IFS+NaHS group compared to the Ado+IFS+PBS group (*p*<0.05). Meanwhile, no significant changes in H_2_S content were observed in the hippocampus of the Adu+IFS+NaHS group compared to the Adu+IFS+PBS group. There was a significant decrease in H_2_S content in the hippocampus of mice in the Adu+IFS+PBS group compared with mice in the Ado+IFS+PBS group (*p*<0.05) (**Figure 3B**). Western blot analysis revealed a significant increase in CBS expression in the hippocampus of the Ado+IFS+NaHS group compared to the Ado+IFS+PBS group (*p*<0.05). However, there was no significant change in CBS expression in the hippocampus of the Adu+IFS+NaHS group compared to the Adu+IFS+PBS group (**Figure 3C**). These findings suggest that NaHS treatment significantly attenuated the IFS-induced reduction in H_2_S content and CBS expression in the hippocampus.

The results of OFT showed that mice in the Ado+IFS+NaHS group spent more time in the central zone compared to mice in the Ado+IFS+PBS group (*p*<0.001) (**Figure 3F**). The total distance traveled by each group of mice did not show any significant differences (**Figures 3D, E**). In contrast, there was no significant difference in the time taken to enter the central zone between the Adu+IFS+NaHS group and Adu+IFS+PBS group. The results of the TST indicated that the immobility time of the Ado+IFS+NaHS group was significantly reduced compared to the Ado+IFS+PBS group (*p*<0.001). In contrast, there was no significant change in the immobility time of the Adu+IFS+NaHS group compared to the Adu+IFS+PBS group. Additionally, the immobility time of mice in the Adu+IFS+PBS group was significantly higher than that of the Ado+IFS+PBS group (*p*<0.05) (**Figure 3G**). Similarly, in the FST, the immobility time of the Ado+IFS+NaHS group was significantly reduced compared to the Ado+IFS+PBS group (*p*<0.001), while no significant difference was observed between the two adult groups (**Figure 3H**). These findings suggest that NaHS treatment effectively improved anxiety and depression-like behavior in adolescence mice but not in adult mice, likely due to insufficient doses of NaHS administered to the adult mice. H&E staining showed that NaHS treatment had no toxic effects on the brain and major peripheral organs (heart, liver, spleen, lungs and kidneys) (**Supplementary Figure S2**).

### 3.4 NaHS treatment improved synaptic plasticity in the hippocampus of adolescent PTSD mice

**Figure 4:**
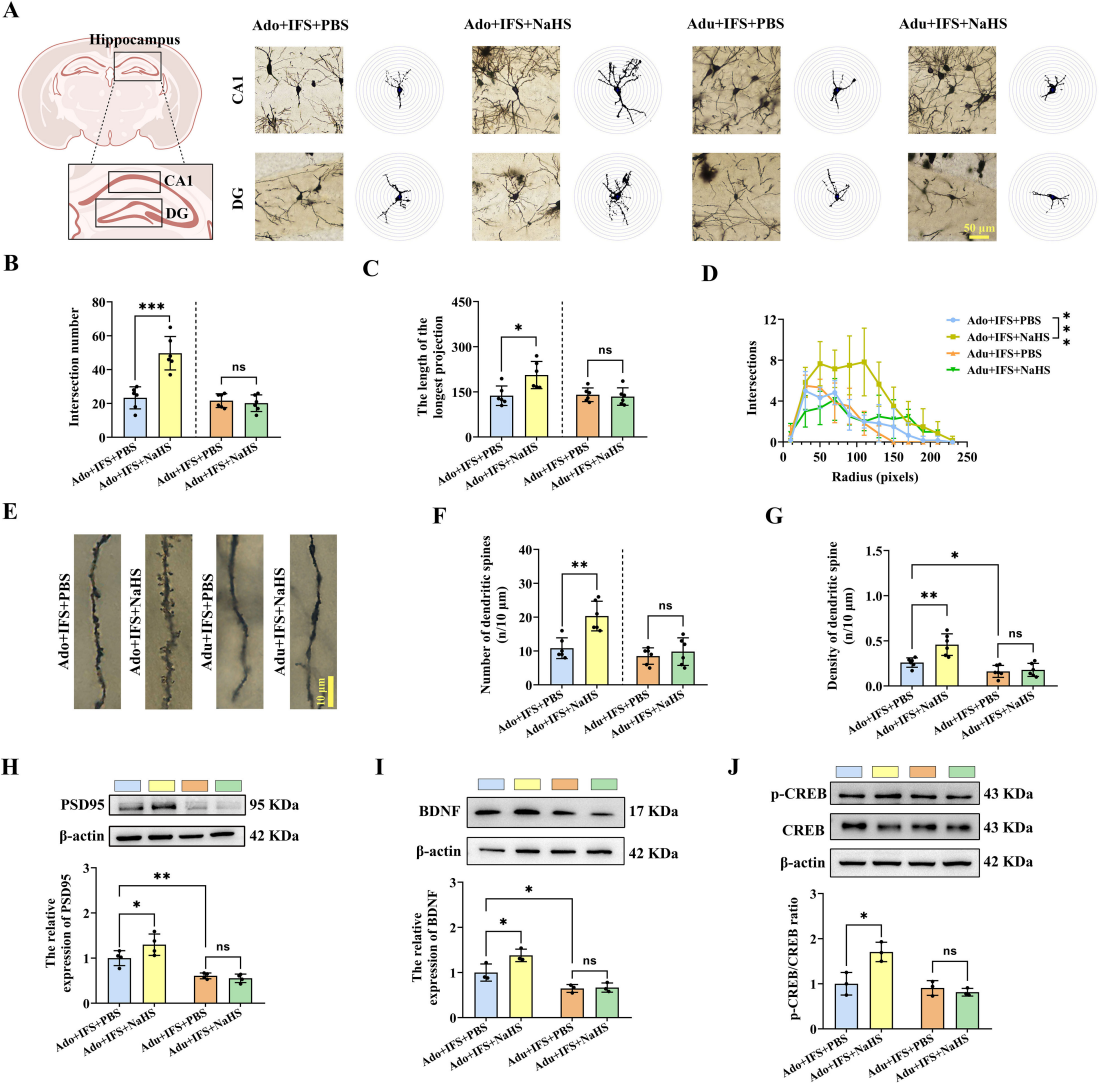
NaHS treatment improved synaptic plasticity in the hippocampus of adolescent PTSD mice. **(A)** Brain atlas showed the locations of CA1 and DG, as well as Golgi staining in these two regions of hippocampus. Scale bars=50 μm. **(B)** The number of intersections of dendritic branches in the hippocampus of adolescent and adult mice. **(C)** The longest length of dendritic branches in the hippocampus of adolescent and adult mice. **(D)** Line graphs of dendritic branch lengths in adolescent and adult mice. **(E)** Golgi staining of dendritic spines in the hippocampus of adolescent and adult mice. Scale bars=10 μm. **(F)** Number of dendritic spines in the hippocampus of adolescent and adult mice. **(G)** Density of dendritic spines in the hippocampus of adolescent and adult mice. **(H)** Western blot analysis of PSD95 in the hippocampus. **(I)** Western blot analysis of BDNF in the hippocampus. **(J)** Western blot analysis of p-CREB/CREB in the hippocampus. N=6 per group in **(A–C, F, G)**; N=4 per group in **(H)**; N=3 per group in **(I, J)** The data were presented as mean ± SD, ns indicates no significant differences, *p<0.05, **p<0.01, ***p<0.001 according to independent samples *t*-test (Ado+IFS+PBS and Adu+IFS+PBS group as a comparison group) in **(B–D, F–J)**.

The dendritic complexity and spine density of CA1 and DG neurons in each group were analyzed using Golgi staining (**Figure 4A**). After NaHS treatment, the number of intersections of dendritic branches was significantly increased in the Ado+IFS+NaHS group compared to the Ado+IFS+PBS group (*p*<0.001). In contrast, there was no significant change in the number of intersections of dendritic branches in the Adu+IFS+NaHS group compared to Adu+IFS+PBS group (**Figure 4B**). In addition, the longest length of dendritic branches was significantly increased in the Ado+IFS+NaHS group compared to the Ado+IFS+PBS group (*p*<0.05). However, NaHS administration did not significantly affect the longest length of dendritic branches in the hippocampal region (**Figure 4C**). The number of intersections of dendritic branches within 250 pixels is shown in a line graph (**Figure 4D**). Moreover, Golgi staining was performed to analyze neuronal dendritic spines in the hippocampus of adolescence and adult mice (**Figure 4E**). The results showed that the number of dendritic spine was significantly increased in the Ado+IFS+NaHS group compared to the Ado+IFS+PBS group (*p*<0.01). In contrast, there was no significant change in the number of dendritic spines in the Adu+IFS+NaHS groups compared to the Adu+IFS+PBS group (**Figure 4F**). Moreover, dendritic spine density was significantly higher in the Ado+IFS+NaHS group compared to the Ado+IFS+PBS group (*p*<0.01), while no significant change was observed in the Adu+IFS+NaHS group compared to the Adu+IFS+PBS group. The dendritic spine density in the hippocampus of mice in the Adu+IFS+PBS group was significantly decreased compared with that in the Ado+IFS+PBS group (*p*<0.01; **Figure 4G**).

Furthermore, PSD95, a key postsynaptic density protein, was significantly increased in the Ado+IFS+NaHS group compared to the Ado+IFS+PBS group (*p*<0.01). However, there was no significant change in PSD95 in the Adu+IFS+NaHS group compared to the Adu+IFS+PBS group (**Figure 4H**). Similarly, NaHS treatment significantly increased BDNF in the hippocampus of the Ado+IFS+NaHS group compared to the Ado+IFS+PBS group (*p*<0.05). In contrast, no significant changes in BDNF were observed in Adu+IFS+NaHS group compared to the Adu+IFS+PBS group. Additionally, BDNF in the Adu+IFS+PBS group was significantly decreased compared to Ado+IFS+PBS (*p*<0.05; **Figure 4I**). Meanwhile, Western blot analysis revealed a significant increase in p-CREB/CREB in the hippocampus of the Ado+IFS+NaHS group compared to the Ado+IFS+PBS group (*p*<0.05). However, there was no significant change in p-CREB/CREB in the hippocampus of the Adu+IFS+NaHS group compared to the Adu+IFS+PBS group (**Figure 4J**).

### 3.5 Increased dose of NaHS improved anxiety and depression-like behavior in adult mice with PTSD and increased CBS/H_2_S levels in the hippocampus

**Figure 5:**
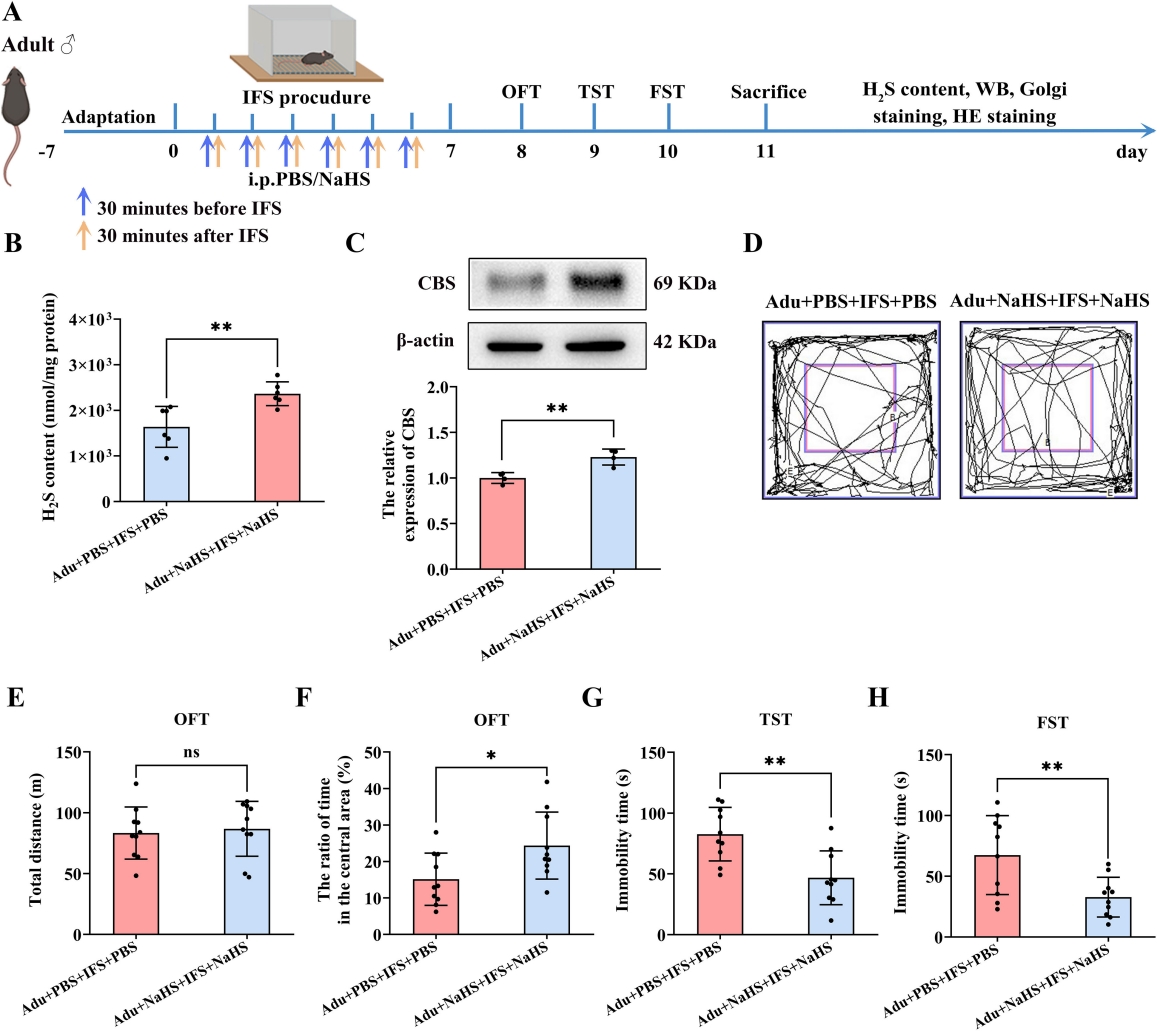
Increasing dose of NaHS improved anxiety and depression-like behavior in adult mice with PTSD and increased CBS/H_2_S levels in the hippocampus. **(A)** Experimental flow chart. The Adu+NaHS+IFS+NaHS group received NaHS (i.p.) 30 min prior to and 30 min after the end of the shocks, while mice in the Adu+IFS group were administered PBS (i.p.) at the indicated time. After completing IFS, OFT was performed on the first day, TST was performed on the second day, and FST was performed on the third day. After mice were sacrificed, the H_2_S content in the hippocampus was tested, and follow-up tests were performed. **(B)** Detection of H_2_S content in the hippocampus of mouse. **(C)** Western blot analysis of CBS in the hippocampus. **(D)** The sample travel pathway in the OFT. **(E)** The total distance in the OFT. **(F)** The ratio of time in the central area in the OFT. **(G)** The immobility time in the TST. **(H)** The immobility time in the FST. N=6 per group in **(B)**, N=4 per group in **(C)**, N=10 per group in **(D–H)**. The data were presented as mean ± SD, ns indicates no significant differences, *p<0.05, **p<0.01 according to independent samples *t*-test (Adu+PBS+IFS+PBS group as a comparison group) in **(B, C, E–H)**.

Anxiety and depression-like behavior, impaired synaptic plasticity in the hippocampus, and decreased p-CREB and BDNF were not significantly reversed in adult PTSD mice treated with a single dose of NaHS. For optimal outcomes, two doses of exogenous H_2_S administration were required for adult PTSD mice (**Figure 5A**). It was found that the H_2_S in the hippocampus of the Adu+NaHS+IFS+NaHS group was significantly elevated compared to the Adu+PBS+IFS+PBS group (*p*<0.01; **Figure 5B**). Western blot analysis revealed a significant increase in CBS in the hippocampus of the Adu+NaHS+IFS+NaHS group compared to the Adu+PBS+IFS+PBS group (*p*<0.01; **Figure 5C**).

Behavioral tests showed no significant difference in the total distance travelled between the two groups (**Figures 5D, E**). However, the Adu+NaHS+IFS+NaHS group took significantly longer to stay in the central zone compared to the Adu+PBS+IFS+PBS group (*p*<0.05; **Figure 5F**). TST results indicated that the immobility time of the Adu+NaHS+IFS+NaHS group was significantly reduced compared to the Adu+PBS+IFS+PBS group (*p*<0.01; **Figure 5G**). Similarly, in the FST, the immobility time was significantly reduced in the Adu+NaHS+IFS+NaHS group compared to the Adu+PBS+IFS+PBS group (*p*<0.01; **Figure 5H**). Histological examination using H&E staining showed that increased dose of NaHS resulted in no toxicity in the brain and major peripheral organs (heart, liver, spleen, lungs and kidneys) (**Supplementary Figure S3**).

### 3.6 Increased dose of NaHS improved synaptic plasticity in the hippocampus in adult post-traumatic stress disorder mice

**Figure 6:**
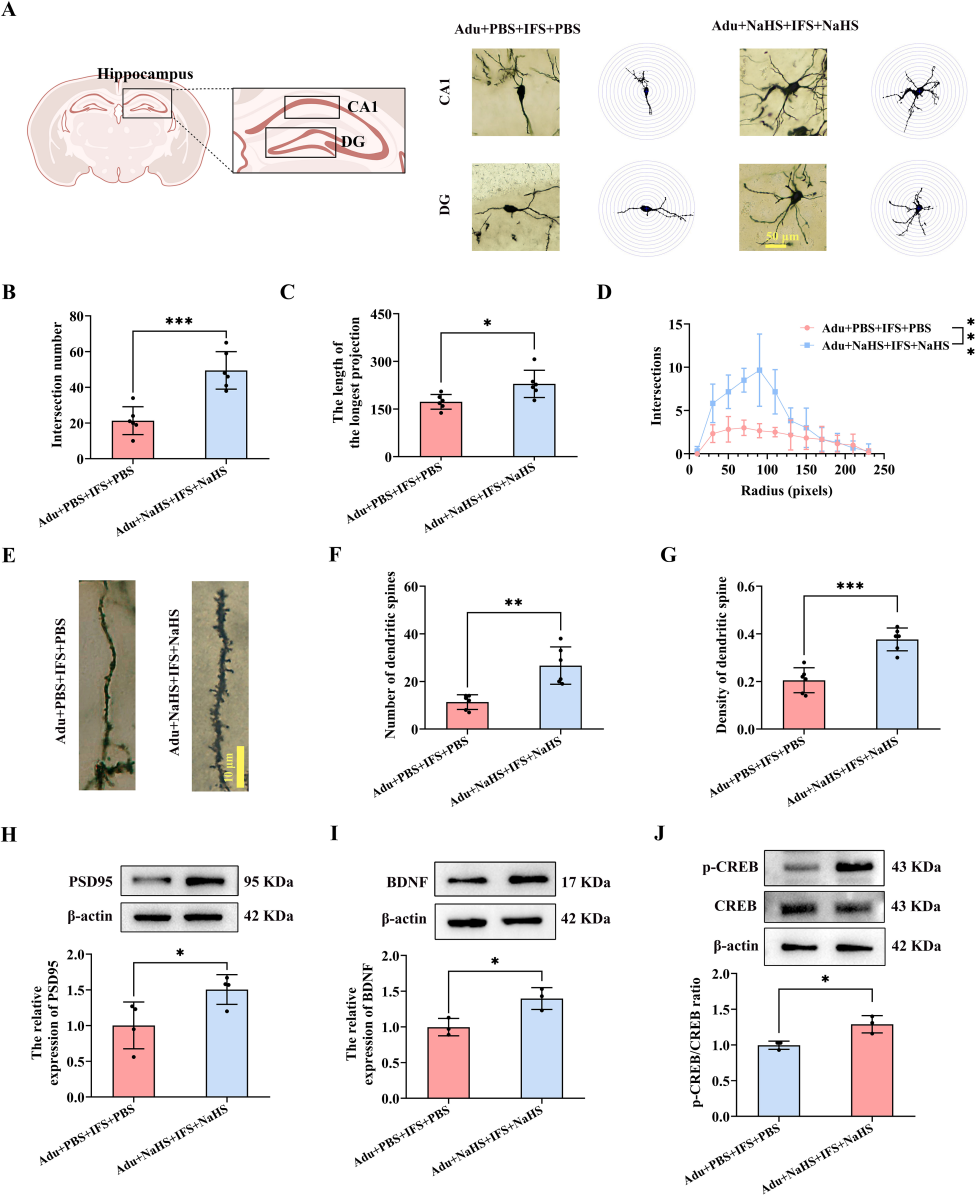
Increasing doses of NaHS improved synaptic plasticity in the hippocampus of adult PTSD mice. **(A)** Brain atlas showed the locations of CA1 and DG, as well as Golgi staining in these two regions of hippocampus. Scale bars=50 μm. **(B)** The number of intersections of dendritic branches in the hippocampus of adult mice. **(C)** The longest length of dendritic branches in the hippocampus of adult mice. **(D)** Line graphs of dendritic branch lengths in adult mice. **(E)** Golgi staining of dendritic spines in the hippocampus of adult mice. Scale bars=10 μm. **(F)** Number of dendritic spines in the hippocampus of adult mice. **(G)** Density of dendritic spines in the hippocampus of adult mice. **(H)** Western blot analysis of PSD95 in the hippocampus. **(I)** Western blot analysis of BDNF in the hippocampus. **(J)** Western blot analysis of p-CREB/CREB in the hippocampus. N=6 per group in **(A–C, F, G)**; N=4 per group in H; N=3 per group in **(I, J)** The data were presented as mean ± SD, *p<0.05, **p<0.01, ***p<0.001 according to independent samples *t*-test in **(B, C, F–I)** and rank sum test in **(J)** (Adu+PBS+IFS+PBS group as a comparison group).

Next, we analyzed the dendritic complexity and spine density of hippocampal CA1 and DG neurons in mice using Golgi staining (**Figure 6A**). The number of intersections of dendritic branches was significantly increased in the Adu+NaHS+IFS+NaHS group compared to the Adu+PBS+IFS+PBS group (*p*<0.001; **Figure 6B**). Furthermore, increased dose of exogenous H_2_S significantly enhanced the longest length of dendritic branches in the hippocampus of the Adu+NaHS+IFS+NaHS group (*p*<0.05; **Figure 6C**). The number of intersections of dendritic branches within 250 pixels was shown in **Figure 6D**. Golgi staining of neuronal dendritic spines in the hippocampus of adult mice was performed (**Figure 6E**). The results showed that the number of dendritic spines was significantly increased in the Adu+NaHS+IFS+NaHS group compared to the Adu+PBS+IFS+PBS group (*p*<0.01; **Figure 6F**). Additionally, administration of exogenous NaHS reversed the decrease in spine density in the Adu+NaHS+IFS+NaHS group compared to the Adu+PBS+IFS+PBS group (*p*<0.001; **Figure 6G**).

In the Adu+NaHS+IFS+NaHS group, PSD95 was significantly increased compared to the Adu+PBS+IFS+PBS group (*p*<0.05; **Figure 6H**). Additionally, increasing the dose of exogenous H_2_S significantly enhanced BDNF expression in the hippocampus of the Adu+NaHS+IFS+NaHS group compared to the Adu+PBS+IFS+PBS group (*p*<0.05; **Figure 6I**). Western blot analysis revealed a significant increase of p-CREB/CREB in the hippocampus of the Adu+NaHS+IFS+NaHS group compared to the Adu+PBS+IFS+PBS group (*p*<0.05; **Figure 6J**).

### 3.7 CBS antibody increased anxiety and depressive-like behavior in adult mice

**Figure 7:**
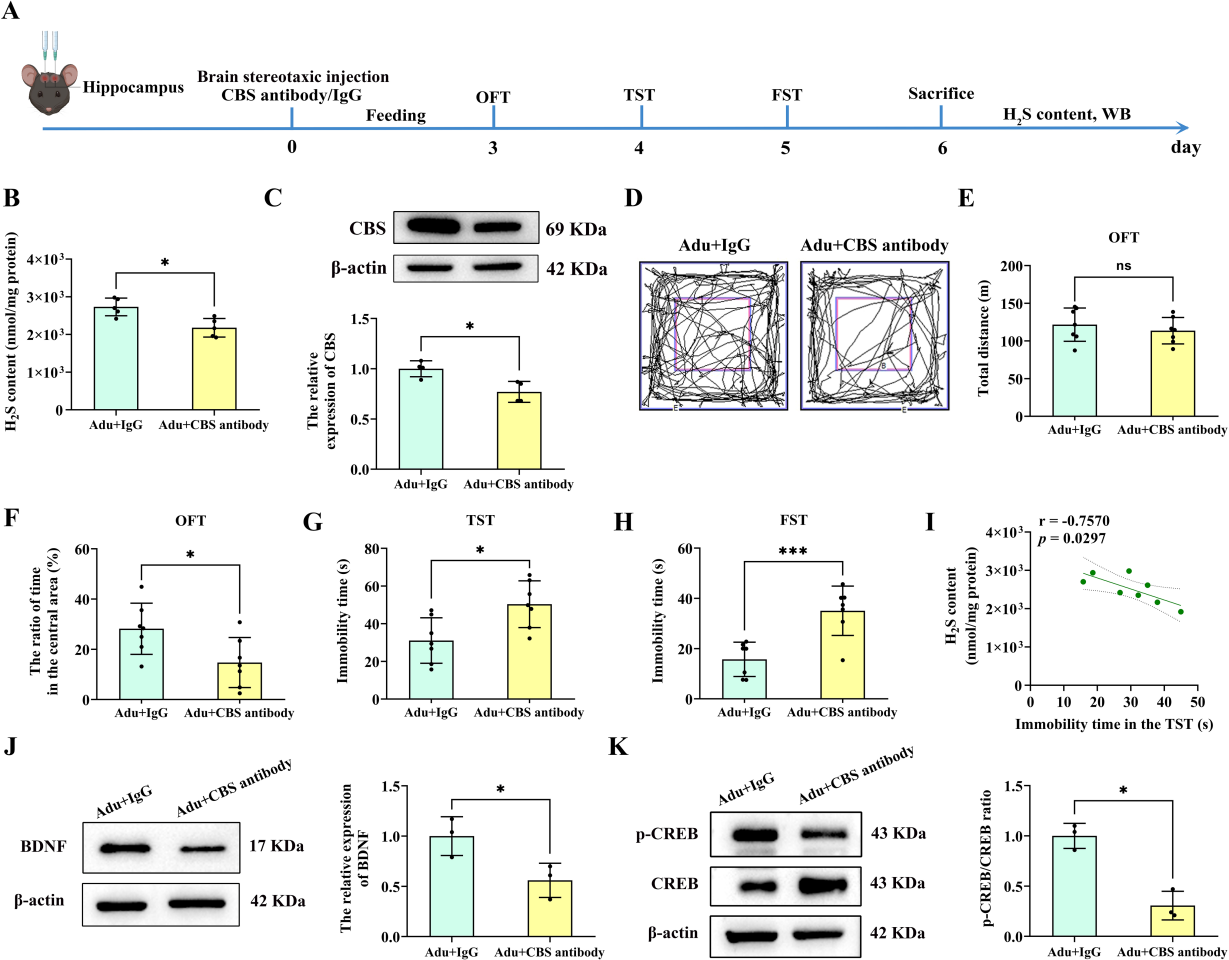
Injection of CBS antibody into the hippocampus of mice led to anxiety and depression-like behavior. **(A)** Experimental flow chart. Mice were injected stereotactically in the hippocampus with CBS antibody or IgG, and behavioral tests were performed 3 days later. OFT was performed on the first day, TST was performed on the second day, and FST was performed on the third day. After the mice were sacrificed, the H_2_S content in the hippocampus was tested, and follow-up tests were performed. **(B)** Detection of H_2_S content in the hippocampus of mice. **(C)** Western blot analysis of CBS in the hippocampus. **(D)** The sample travel pathway in the OFT. **(E)** The total distance in the OFT. **(F)** The ratio of time in the central area in the OFT. **(G)** The immobility time in the TST. **(H)** The immobility time in the FST. **(I)** Correlation analysis of H_2_S content in the hippocampus and the immobility time in the TST. **(J)** Western blot analysis of BDNF in the hippocampus. **(K)** Western blot analysis of p-CREB/CREB in the hippocampus. N=6 per group in B, F and G; N=4 per group in C; N=10 per group in E and **(I)** N=3 per group in J and **(K)** The data were presented as mean ± SD, *p<0.05, ***p<0.001 according to independent samples *t*-test (Adu+IgG group as a comparison group) in **(B, C, E–H, J, K)** correlation analysis in I.

To further verify whether the CBS/H_2_S pathway is involved in PTSD-like behavior in mice, the CBS antibody was stereotactically injected into the mouse hippocampus to neutralize CBS expression (**Figure 7A**). H_2_S contents in the hippocampus of the Adult+CBS antibody group was significantly decreased compared to the Adult+IgG group (*p*<0.01; **Figure 7B**). Western blot analysis also revealed a significant decrease in CBS in the hippocampus of the Adult+CBS antibody group compared to the Adult+ IgG group (*p*<0.05; **Figure 7C**). Behavioral results showed that the total distance traveled by the mice did not change (**Figures 7D, E**). However, the Adult+CBS antibody group spent a shorter time to stay in the central zone compared to the Adult+IgG group (*p*<0.05; **Figure 7F**) In both the TST and the FST, the immobility times of the Adult+CBS antibody group were significantly increased compared to those of Adult+IgG mice (*p*<0.05; *p*<0.001; **Figures 7G, H**). Additionally, a strong correlation was observed between hippocampal H_2_S content and immobility time in the TST (**Figure 7I**).

Furthermore, BDNF in the hippocampus of the Adult+CBS antibody group was significantly decreased compared to the Adult+IgG group (*p*<0.05; **Figure 7J**). Similarly, Western blot analysis revealed a significant decrease in p-CREB/CREB in the hippocampus of the Adult+CBS antibody group compared to the Adult+IgG group (*p*<0.05; **Figure 7K**). These findings suggest that neutralizing CBS expression in the hippocampus led to PTSD-like behaviors and downregulated these key synaptic plasticity related proteins.

## 4 Discussion

In the present study, we found that adult PTSD mice exhibited more pronounced decrease in H_2_S content and CBS expression in the hippocampus, which were associated with anxiety and depression-like behavior compared with adolescence PTSD mice. Administration of exogenous H_2_S (one dose for one day) significantly improved anxiety and depression-like behavior and synaptic plasticity deficits, and activated the CREB/BDNF signaling pathway in the hippocampus of adolescence PTSD mice. We also found that increased doses of exogenous H_2_S administration could improve anxiety and depression-like behavior, synaptic plasticity deficits, and activate the CREB/BDNF signaling pathway, as well as increase H_2_S levels in the hippocampus. Moreover, similar anxiety and depression-like behavior were observed in mice after injection of CBS antibodies into the hippocampus. These results suggest that the H_2_S/CBS system had an age-dependent effect on IFS-induced PTSD and might involve in synaptic plasticity via activation of the CREB/BDNF signaling pathway after IFS exposure.

Individuals with PTSD often experience an increased prevalence of age-related diseases. To elucidate the stress vulnerability across different ages, it is essential to examine the effects of the same types of stressors in both adolescence and adult populations. However, studies of this nature have been limited, and the existing research has yielded inconsistent behavioral results. Some studies have found that adolescent rodents and humans exhibit increased social activity, risk-taking behavior, and impulsivity compared to other age groups (27, 28). For example, mice exposed to social instability stress during adolescence demonstrate greater stress vulnerability compared to adult mice subjected to the same stressors (29). In contrast, some studies have reported that adolescents are more resilient to stress-induced deleterious effects compared to adults. For example, following resident-intruder social stress, defensive burying behavior was found to be decreased in adult mice compared to adolescent mice (30). Moreover, the same types of stressors induced depression-like behaviors and decreased neurogenesis (31), as well as impaired learning and memory function (32, 33) in adult mice but not in adolescent mice. Furthermore, corticosterone treatment resulted in increased anxiety levels and cognitive impairment in adult animals, while adolescent individuals exhibited the opposite behavioral changes (34). Our study found that the same types of stressor exposure produced a series of behavioral and neurobiological effects with striking age differences. Adult PTSD mice exhibited greater anxiety and depression-like behavior and synaptic plasticity deficits, as well as reduced p-CREB and BDNF compared with adolescent PTSD mice. These results indicate that PTSD may render adult individuals less capable of defending themselves against unexpected stimuli compared with adolescent individuals. Further studies are needed to evaluate whether these differing effects on adolescents and adult extend to other contexts.

H_2_S has been recognized as the third gasotransmitter, following nitric oxide and carbon monoxide, and it exerts important biological effects in the body (35). H_2_S is endogenously synthesized from l-cysteine by several enzymes, including CSE, CBS, 3-MST and cysteine aminotransferase (CAT) (36). In the CNS, endogenous H_2_S is mainly produced from L-cysteine catalyzed by CBS (37).

CBS is the main contributor to the production of H_2_S in the brain (38), especially in astrocytes (39). Depletion of CBS in brain astrocytes leads to mitochondrial dysfunction and oxidative stress. Supplementation with H_2_S, on the other hand, alleviates this impairment (39). H_2_S levels and CBS expression were decreased in the hippocampus of depressed mice, whereas aerobic exercise promoted endogenous H_2_S production as well as an increase in CBS expression and contributed to the improvement of hippocampal neurological function and antidepressant effects (40). CSE is the major H_2_S-producing enzyme in the vasculature, liver and kidney (41), and CSE is also expressed in smooth muscle and endothelial cells as well as in perivascular adipose tissue (42). It has been shown that knockout of CSE in mice resulted in reduced endogenous H_2_S levels and hypertension as well as reduced endothelium-mediated vasodilation in mice (43). Meanwhile, genetic deficiency of CSE exacerbates atherosclerosis in murine models, while exogenous H_2_S administration selectively attenuates these pro-atherogenic effects (41). In the kidney, on the other hand, increasing endogenous H_2_S content and CSE expression by administration of exogenous H_2_S was able to reverse renal fibrosis and thus improve diabetes (44). The CSE/H_2_S pathway is also downregulated in a mouse model of high-fat diet-induced nonalcoholic fatty liver disease (45). In addition, 3-MST is localized to both the mitochondria and the cytosol (46). It has been demonstrated that 3-MST plays an important role in mitochondrial function and ATP production in hepatocytes (47). In addition, 3-MST/H_2_S attenuation reduced mitochondrial respiration and mitochondrial ATP production and perturbed several pathways in the endothelial cell metabolome (48). In conclusion, under physiological conditions, CBS, CSE and 3-MST play important roles in H_2_S synthesis in different tissues, respectively.

In addition, studies have reported that administration of exogenous NaHS can enhance CBS protein levels (49). NaHS is one of the donors of H_2_S and H_2_S in NaHS solution is stable (11), therefore we chose NaHS as the H_2_S donor in this study. NaHS can be hydrolyzed into Na^+^ and HS^–^, the latter being able to bind H^+^ in the body to produce H_2_S (11). For example, exogenous H_2_S restored CBS and CSE enzyme expression in the hypothalamus 28 days after traumatic brain injury (50). We found that H_2_S content in the hippocampus was significantly increased in the Ado+IFS+NaHS group compared to the Adu+IFS+PBS group. Meanwhile, no significant changes in H_2_S content were observed in the hippocampus of the Ado+IFS+NaHS group compared to the Adu+IFS+PBS group. Previous studies have shown that H_2_S synthase expression is regulated by transcription factors, such as nuclear factor erythroid-2-related factor 2 (Nrf2) (51). It is well known that H_2_S regulates Nrf2 activity through Kelch-like ECH-associated protein 1 (Keap1) sulfhydration, thereby reducing oxidative stress (52). Thus, it is possible that exogenous H_2_S binds to Keap1 and induces Nrf2 dissociation, which in turn enhances Nrf2 nuclear translocation and promotes the expression of CBS and CSE enzymes (52). Another study demonstrated that NaHS significantly increased the expression of CBS and production of endogenous H_2_S after intracerebral hemorrhage injury (53).

NaHS is one of the exogenous donors of H_2_S. In determining the appropriate dose of NaHS, we carefully referenced previous studies that have established the efficacy and safety of dose in animal models of stress-related disorders. A study reported a dose-dependent increase in SMI-32–positive neurons death starting at 100 μM (54). Moreover, to evaluate the selectivity of the H_2_S-mediated toxicity to motor neurons, the report examined the survival of the primary spinal GABAergic neurons. NaHS exposure was toxic to GABAergic neurons from a concentration as high as 600 μM (54). Specifically, we adopted a dose of 5.6 mg/kg/day, which was administered 30 minutes after each stress induction session. This dose is therapeutically effective in a variety of disease models as previously reported in the literature. For example, this dose of NaHS is neuroprotective in a vascular dementia model (55). In chronic renal failure, this dose of NaHS exerts anti-inflammatory and anti-apoptotic effects (56). This dose of NaHS was also able to protect against damage caused by myocardial ischemia-reperfusion (57). Meanwhile, several reports have shown that this dose of NaHS plays a therapeutic role in depression (16, 17). In addition, Kitti Göntér et al. reported that compared with the untreated or vehicle group, dimethyl trisulfide at a dose of 60 mg/kg treatment reduced the distance traveled or duration of activity of OFT in mice, but dimethyl trisulfide at a dose of 50 mg/kg did not affect the distance traveled or duration of activity in mice (58). The open field experiment is one of the effective methods to detect the potency of drugs and the individual condition of mice. In our pre-experiment, the administration of 5.6 mg/kg of NaHS was not affect total distance of OFT in control mice and IFS mice. Therefore, we chose a dose of 5.6 mg/kg/day for our experiment.

Some studies have shown that treatment with H_2_S may become a promising intervention to prevent, delay, or reverse aging (39). For instance, the function of senescent bone marrow mesenchymal stem cells can be rescued using H_2_S via the calmodulin-NFAT1 signaling pathway (59). In the present study, we first demonstrated that the decrease in H_2_S content and CBS expression was more pronounced in the hippocampus of adult mice compared to adolescent mice after exposure to PTSD stimuli. This difference led to the inability of a single dose of H_2_S treatment to reverse PTSD-like behavior, H_2_S contents and CBS expression in adult PTSD mice. Therefore, for optimal outcomes, we administered two doses of exogenous H_2_S to adult PTSD mice. Furthermore, we found that increasing dose of exogenous H_2_S administration could improve anxiety and depression-like behavior, synaptic plasticity deficits, p-CREB/CREB and BDNF, as well as increase H_2_S levels in the hippocampus. These results indicate that age-dependent variations in hippocampal CBS/H_2_S levels following IFS contribute to divergent behavioral and synaptic plasticity outcomes between adolescent and adult mice.

Synaptic plasticity refers to the brain’s ability to undergo flexible adjustment and reorganization in response to changes in the neuro-environment. This capacity allows the brain to adapt to altered neural conditions through dynamic structural and functional modifications (60). Previous studies have shown that traumatic stresses can reduce structural synaptic plasticity in the hippocampus of adolescent rats and induce PTSD-like behaviors (22). Consistent with these findings, our study demonstrated that the IFS procedure reduced structural synaptic plasticity in the hippocampus of both adolescent and adult mice compared to control mice. Moreover, this reduction was more pronounced in the hippocampus of adult mice than in adolescent mice following PTSD stimuli. BDNF, an important protein associated with synaptic plasticity, is typically downregulated in patients with PTSD (61). Conversely, increasing BDNF levels has been shown to enhance neural plasticity and reverse affective disorders (62). CREB is a key transcriptional regulator involved in neuronal differentiation, synaptic plasticity, learning and memory, and it regulates the expression of BDNF. In the brain, it plays a significant role in hippocampal neuroplasticity, long-term memory formation, dendritic growth, and neurogenesis. CREB function is mainly regulated by phosphorylation of Ser133 (63). In the brain, CREB activation by phosphorylation at Ser133 and the recruitment of transcription cofactors such as CREB binding protein (CBP) is a critical step for the formation of memory. Phosphorylated CREB, as activated state of CREB, stimulates expression of BDNF and promotes synaptic plasticity and neurogenesis (64, 65). It is reported that during chronic stress damage in neurons during adulthood, BDNF helps to protect neurons, regulates calcium concentration and reduces the necrosis and apoptosis in hippocampal neurons, positively affects neuronal plasticity (66). ω-3PUFAs could increase CREB phosphorylation on ser 133 cite to regulate the expression of multiple proteins involved in memory (67). In addition, BDNF induces phosphorylation of CREB at Ser-133, and p-CREB interacts with other related proteins and is involved in the regulation of mood and memory (68). In summary, CREB/BDNF signaling pathways may improve the molecular basis of neurogenesis, synaptic plasticity, memory, and emotion (69).

A previous study reported that the protein levels of p-CREB and BDNF were decreased in the hippocampus of a PTSD animal model (70), which is consistent with our findings. In our study, the levels of PSD95, p-CREB/CREB, and BDNF protein expression were significantly decreased in the hippocampus of mice subjected to IFS compared to control mice. Additionally, we observed that the decreases in p-CREB/CREB and BDNF protein levels in response to stress were age-dependent. Compared to adolescent IFS mice, adult IFS mice exhibited more pronounced reductions in PSD95, p-CREB/CREB, and BDNF in the hippocampus. Moreover, the levels of these protein were significantly improved in the hippocampus of adolescent IFS mice treated with NaHS (ado+IFS+NaHS) compared to those treated with PBS (ado+IFS+PBS). Similarly, these protein levels were significantly increased in the hippocampus of adult IFS mice treated with NaHS (adu+NaHS+IFS+NaHS) compared to those treated with PBS (adu+IFS+PBS). These improvements were associated with restored dendritic morphology and spine density. In conclusion, CBS/H_2_S may ameliorate anxiety and depression-like behaviors by activating the CREB/BDNF signaling pathway in the hippocampus of mice exposed to IFS. Conversely, injection of the CBS antibody directly decreased CREB/BDNF signaling pathway and exacerbated PTSD-like behaviors. Additionally, we found a strong correlation between hippocampal H_2_S levels and PTSD-like behaviors in mice. These results suggest that CBS/H_2_S modulates age-related differences in stress response and synaptic plasticity through the CREB/BDNF signaling pathway.

Although our study focused on mice, it is important to consider the translational implications for humans. Many studies have shown that H_2_S concentrations detected in the brain of mammals such as humans, rats, and cattle range from 50 to 160 μM (20, 71–74). This enhances the translational significance of our findings. Thus, our findings in mouse models can be used to understand H_2_S dynamics in human neurophysiology and pathology. This parallel is particularly important for the development of therapeutic strategies targeting H_2_S pathways, as it increases the likelihood that interventions that are effective in mice will be successfully translated into human. In addition, the conserved nature of H_2_S levels supports the use of mouse models in preclinical studies of neurological disorders involving H_2_S dysregulation, which has the potential to accelerate the development of novel therapeutic approaches for diseases such as Alzheimer’s disease, Parkinson’s disease, and stroke.

The present study acknowledges several limitations. Firstly, though our experiment demonstrated that exogenous H_2_S administration reversed the decrease in hippocampal synaptic plasticity and p-CREB/CREB and BDNF in mice, we did not delve deeply into the specific underlying mechanisms. This gap highlights a direction for future research. Secondly, numerous studies have shown that the hippocampus, amygdala, and medial prefrontal cortex are all involved in modulating PTSD (75). In our study, we focused on the hippocampus due to its critical role in fear, episodic and contextual learning, and memory processes associated with PTSD symptomatology (76). Indeed, significant reductions in hippocampal volume have been observed in both human PTSD patients (77) and PTSD mice (78). However, we acknowledge that other brain regions, such as the amygdala and medial prefrontal cortex, may also play important roles in the effect of H_2_S/CBS on age-dependent PTSD. Future studies are needed to identify H_2_S/CBS-dependent neurobiological changes in each of these regions and to examine how these changes relate to behavior.

In conclusion, our study highlights the age-dependent effects of stress on PTSD-like behaviors and synaptic plasticity, which are mediated by differential changes in hippocampal H_2_S content and CBS expression following exposure to IFS. Adult PTSD mice exhibited more severe behavioral and neurobiological deficits than adolescents, necessitating tailored therapeutic approaches, such as multiple H_2_S doses, for optimal outcomes. These findings underscore the potential of H_2_S-based interventions in addressing age-related vulnerabilities to stress and PTSD, providing a foundation for future research into the mechanistic and translational aspects of H_2_S signaling in the brain.

## Data Availability

The original contributions presented in the study are included in the article/**Supplementary Material**. Further inquiries can be directed to the corresponding authors.
