# Supplementary material for: Age-dependent effects of H2S on post-traumatic stress disorder in adolescent and adult mice
Source: Front Psychiatry. 2025 Jun 9;16:1546737. doi: 10.3389/fpsyt.2025.1546737 (PMC12183304; doi:10.3389/fpsyt.2025.1546737)
Supplement: Supplementary file 1 [file DataSheet1.pdf]

Figure legends

Figure S1 IFS had no toxicity on brain and peripheral major organs in adolescent and adult mice

At the end of the behavioral tests, mice were sacrificed, the brains and major organs were removed and then fixed in 4% paraformaldehyde. Hematoxylin and Eosin (H&E) staining of brain (Scale bars=100 μm) and peripheral major organs (Scale bars=50 μm) in control or PTSD mice.

Figure S2 NaHS treatment had no toxicity on brain and peripheral major organs in adolescent and adult mice

At the end of the behavioral tests, mice were sacrificed, the brains and major organs were removed and then fixed in 4% paraformaldehyde. H&E staining of brain (Scale bars=100 μm) and peripheral major organs (Scale bars=50 μm) in PTSD or NaHS-treatment mice.

Figure S3 Increasing dose of NaHS treatment had no toxicity on brain and peripheral major organs in adult mice

At the end of the behavioral tests, mice were sacrificed, the brains and major organs were removed and then fixed in 4% paraformaldehyde. H&E staining of brain (Scale bars=100 μm) and peripheral major organs (Scale bars=50 μm) in PTSD or two doses of exogenous H_2S treatment mice.

Table S1 Primary antibody information

Antibody	Company	Catalog number
CBS	Proteintech group (Rosemont, IL, USA)	14787-1-AP
CREB	Cell Signaling Technology, Inc. (Boston, MA, USA)	9198S
BDNF	Proteintech group (Rosemont, IL, USA)	17465-1-AP
PSD95	Proteintech group (Rosemont, IL, USA)	20665-1-AP
p-CREB	Cell Signaling Technology, Inc. (Boston, MA, USA)	9197S
β-Actin	ZSGB-BIO (Beijing, China)	TA-09
