# Supplementary figures and images for: Age-dependent effects of H2S on post-traumatic stress disorder in adolescent and adult mice
Source: Front Psychiatry. 2025 Jun 9;16:1546737. doi: 10.3389/fpsyt.2025.1546737 (PMC12183304; doi:10.3389/fpsyt.2025.1546737)

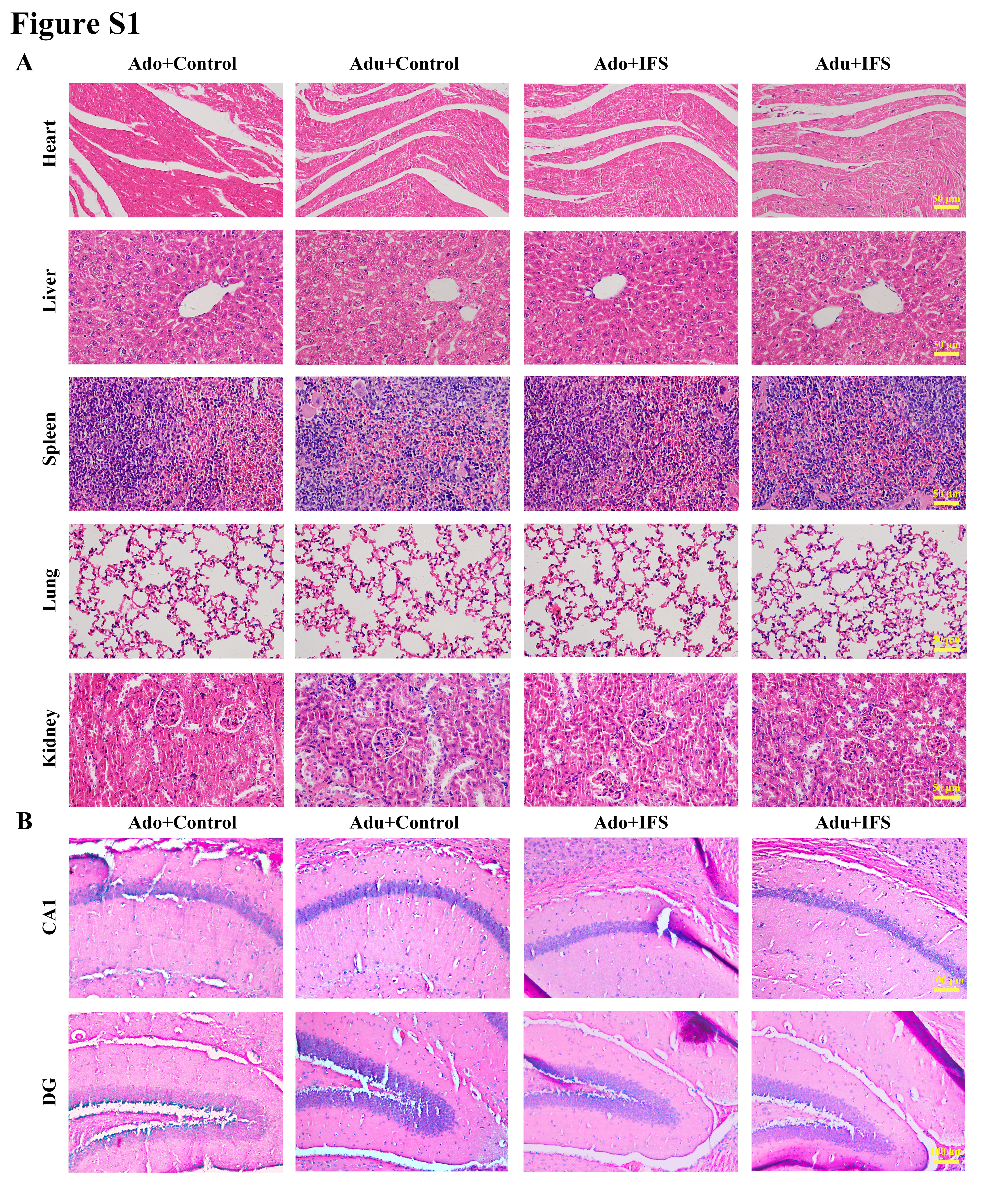

Supplement: Supplementary file 3 [file Image1.jpeg]

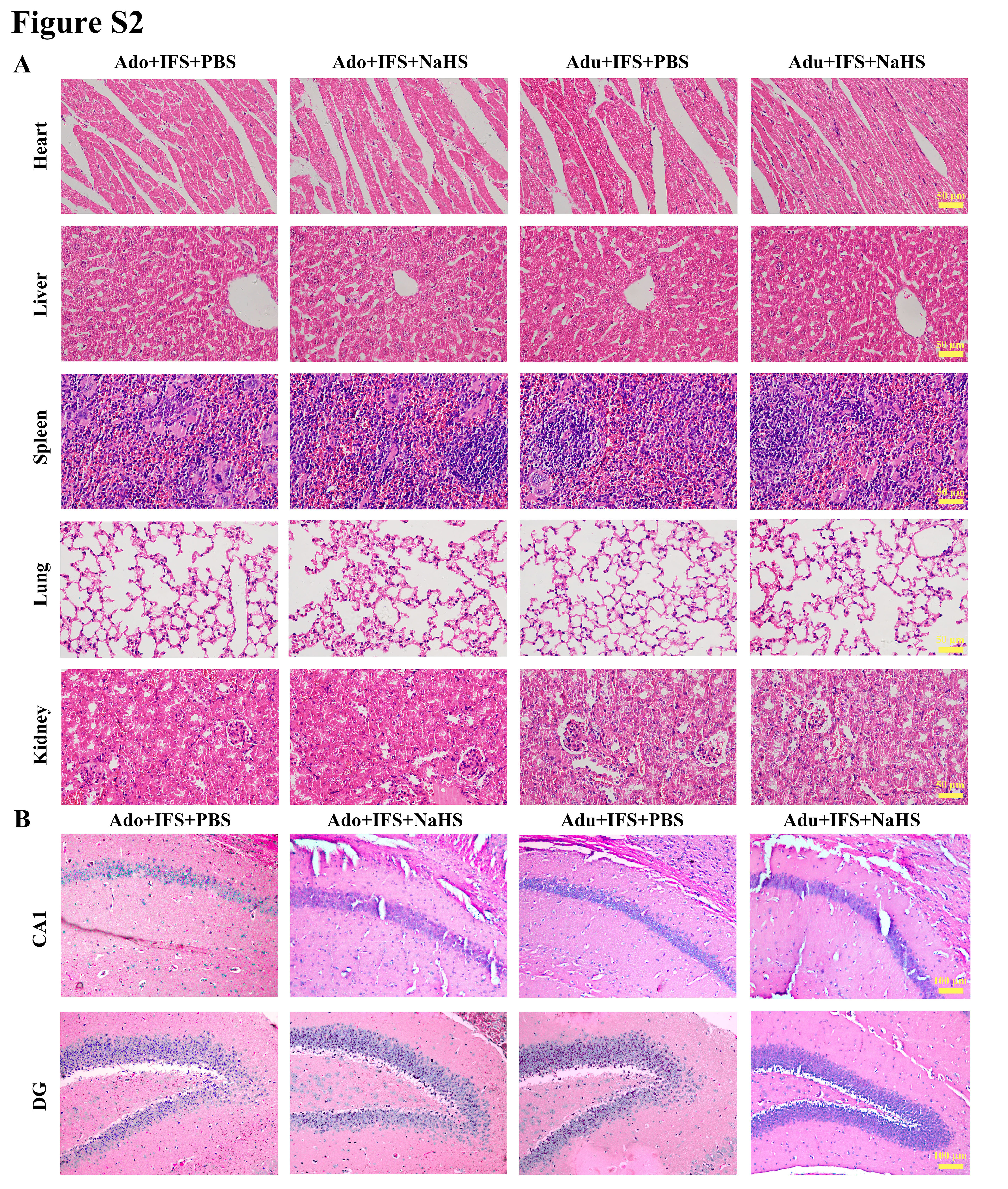

Supplement: Supplementary file 4 [file Image2.jpeg]

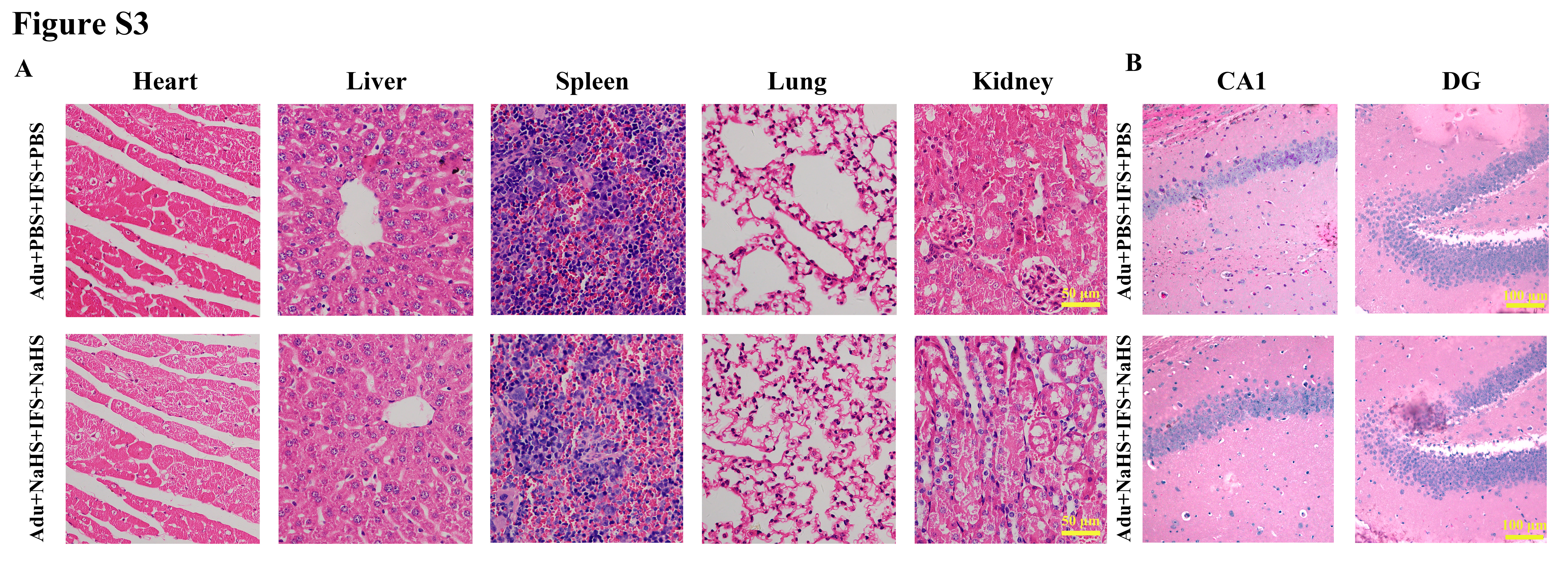

Supplement: Supplementary file 5 [file Image3.jpeg]
